# Metabolomic Profiling of Faecal Extracts from *Cryptosporidium parvum* Infection in Experimental Mouse Models

**DOI:** 10.1371/journal.pone.0077803

**Published:** 2013-10-18

**Authors:** Josephine S. Y. Ng Hublin, Una Ryan, Robert Trengove, Garth Maker

**Affiliations:** 1 School of Veterinary and Life Sciences, Murdoch University, Murdoch, Western Australia, Australia; 2 Murdoch University Separation Science and Metabolomics Laboratory, Murdoch University, Murdoch, Western Australia, Australia; 3 Metabolomics Australia, Murdoch University Node, Perth, Western Australia, Australia; University at Buffalo, United States of America

## Abstract

Cryptosporidiosis is a gastrointestinal disease in humans and animals caused by infection with the protozoan parasite *Cryptosporidium*. In healthy individuals, the disease manifests mainly as acute self-limiting diarrhoea, but may be chronic and life threatening for those with compromised immune systems. Control and treatment of the disease is challenged by the lack of sensitive diagnostic tools and broad-spectrum chemotherapy. Metabolomics, or metabolite profiling, is an emerging field of study, which enables characterisation of the end products of regulatory processes in a biological system. Analysis of changes in metabolite patterns reflects changes in biochemical regulation, production and control, and may contribute to understanding the effects of *Cryptosporidium* infection in the host environment. In the present study, metabolomic analysis of faecal samples from experimentally infected mice was carried out to assess metabolite profiles pertaining to the infection. Gas-chromatography mass spectrometry (GC-MS) carried out on faecal samples from a group of *C. parvum* infected mice and a group of uninfected control mice detected a mean total of 220 compounds. Multivariate analyses showed distinct differences between the profiles of *C. parvum* infected mice and uninfected control mice,identifying a total of 40 compounds, or metabolites that contributed most to the variance between the two groups. These metabolites consisted of amino acids (n = 17), carbohydrates (n = 8), lipids (n = 7), organic acids (n = 3) and other various metabolites (n = 5), which showed significant differences in levels of metabolite abundance between the infected and uninfected mice groups (p < 0.05). The metabolites detected in this study as well as the differences in abundance between the *C. parvum* infected and the uninfected control mice, highlights the effects of the infection on intestinal permeability and the fate of the metabolites as a result of nutrient scavenging by the parasite to supplement its streamlined metabolism.

## Introduction

Cryptosporidiosis, a gastroenteric disease characterized mainly by diarrheal illnesses in humans and mammals is caused by infection with the protozoan parasite *Cryptosporidium*. The disease, though mainly self-limiting in those that are immunocompetent, can be chronic and lead to severe dehydration with terminal results in those that are immunocompromised [1]. Children, particularly those age five years or less are most susceptible to cryptosporidiosis where chronic infections have been shown to impair growth and cognitive development. Treatment options for cryptosporidiosis are limited, with the current therapeutic nitazoxanide only efficacious in those that are immunocompetent, as its efficacy relies on an appropriate host immune response [2]. 

More than 10 different *Cryptosporidium* species have been found to infect humans, although most sporadic cases of human cryptosporidiosis are caused by the anthroponotic *C. hominis* and the zoonotic *C. parvum* [3]. Transmitted directly or indirectly through the oral faecal route, *Cryptosporidium* has a low infectious dose of between 1-10 oocysts with infected humans and cattle reported to shed up to 10^8^ oocyst in a single bowel movement [4]. In order to effectively control and understand the aetiology of *Cryptosporidium*, diagnosis of the parasite is important. Although more sensitive molecular tools are available and widely used for the detection of *Cryptosporidium* [3], due to the costs involved, routine diagnosis of *Cryptosporidium* in most pathology laboratories still relies on microscopy, which lacks specificity and sensitivity, particularly when oocyst numbers are low [5,6]. 

The field of metabolomics provides a novel approach to examine parasites and interactions with their host through the analyses of the entire metabolite or small molecule (<1 kDa) composition of a biological sample. Perturbations in the profiles of these metabolites reflect changes to cellular regulation and physiological processes, as a result of these parasitic infections [7–11]. Although still in its infancy in *Cryptosporidium* research, metabolomics is complementary to the current knowledge of parasite biology and provides an avenue for biomarker discovery, drug targets and improved diagnostics. 

A recent preliminary metabolomics study on *Cryptosporidium* developed a faecal metabolite extraction method for untargeted gas chromatography-mass spectrometry (GC-MS) analysis using *Cryptosporidium* positive and negative human faecal samples [12]. In that study, faecal metabolite profiles of cryptosporidiosis positive patients could be differentiated from cryptosporidiosis negative patients, suggesting that metabolic homeostasis and intestinal permeability were affected as a result of the infection [12]. However, as the metabolome is sensitive to external perturbations, it was difficult to examine the extent to which the changes to the human faecal metabolites observed were attributed to infection with *Cryptosporidium* or to other factors such as age, diet and immune susceptibility etc. which may differ between patients. Hence the aim of the present study was to carry out a more controlled metabolomics analysis of faecal metabolite profiles using experimentally infected mice, to better characterise metabolic changes associated with *Cryptosporidium* infection.

## Materials and Methods

### Experimental infection in mice

The *C. parvum* (S26) isolate used in the present study to infect mice was originally obtained from a naturally infected calf from the Institute of Parasitology, University of Zurich, Switzerland and was passaged through mice at Murdoch University using methods described by Meloni and Thompson [13]. Briefly, 1-day old neonatal Swiss mice (n = 10) obtained from the Animal Resource Centre (Perth, Western Australia) were inoculated individually with approximately 100,000 oocysts. As all the faecal pellets from this litter were required to produce consistent metabolomics data, a second litter (n=10) was inoculated as described above and the mice were confirmed as positive for *Cryptosporidium* by microscopy. A third litter (n=10) was not inoculated and used as an uninfected control for the experiment. The mice were then returned to their dams and euthanized 8 days post-inoculation by CO_2_ exposure. Faecal samples from each mouse were collected directly from the rectum into 1.5 ml microcentrifuge tubes, placed in liquid nitrogen then on dry ice to arrest metabolism. Samples were then stored at -80°C until analysis. Mice infection and metabolomics experiments were repeated twice for reproducibility. All research on animals was conducted under Murdoch University animal ethics permit no: R2351/10.

### Chemicals

All chemicals were purchased from Sigma Aldrich (Australia) at a purity of > 99% unless otherwise noted. Methanol and *n*-hexane (> 95%) were purchased from LabScan (Australia). 

### Metabolite extraction and sample preparation

Metabolite extraction was carried out as described in Ng et al. [12], with minor modifications, to allow for the small amount of faeces produced by each mouse. Briefly, faecal samples from each mouse were freeze-dried overnight to remove all moisture and their subsequent dry weight measured. Methanol containing the internal standard ribitol (10 µg/ml) was added to each sample to a final concentration of 50 mg dry faeces/ml, vortexed until completely homogeneous and then centrifuged at 2530 x *g* at 10°C for 15 minutes. From each sample, a 50 µl aliquot of the extract was dried in a rotary vacuum concentrator and stored at -80°C until derivatisation. Prior to analysis, methoximation and trimethysilyl derivatisation was carried out as described in Ng et al. [12].

### GC-MS instrumentation and analysis

Gas chromatography (GC)-mass spectrometry (MS) analysis was performed on 1 µL of each derivatised sample in splitless mode on an Agilent 6890 gas chromatograph coupled to an Agilent 5973N mass spectrometer as described in Ng et al. [12]. An Agilent FactorFour VF-5ms capillary column (ID = 0.25 mm, Df = 0.25 µm) measuring 30 m with 10 m EZ-Guard was used for separation. Helium was used as a carrier gas with retention time locked to elute mannitol-TMS at 30.6 minutes. Initial oven temperature was set to 70°C, with a temperature ramp set to increase at a rate of 1°C/min for 5 minutes, and subsequently 5.63°C/min to 300°C, holding for 10 minutes. The injector was set at 230°C, the transfer line to the MS at 300°C and the MS ion source at 230°C. The mass spectrometer was set to scan the range *m/z* 45 to 600 at 1 scan per second. 

### Data processing and statistical analysis

The GC-MS data files were deconvoluted and normalised to the internal standard, ribitol. Compounds were matched against an in-house library or reference compounds from the National Institute of Standards and Technology (NIST) (http://www.nist.gov) using AnalyzerPro v2.7.0 (SpectralWorks, UK). Data was then exported to The Unscrambler® X (CAMO, Norway) where principal component analysis (PCA) of the data was performed. Univariate statistical analysis of the metabolites identified was carried out and an unpaired T-test performed to compare metabolite intensities between the infected and non-infected groups with p ≤ 0.05 considered statistically significant. 

## Results

### Analysis of the metabolites identified in faecal samples from *C. parvum* infected mice and uninfected mice

GC-MS analysis of faecal samples from 8-day old *C. parvum* infected and uninfected mice (n =20) detected approximately 220 compounds. A total of 101 of the compounds detected were matched against the in-house library and reference database (Table S1), of which, 87 were identified to belong to various classes, including amino acids, carbohydrates, organic acids, amines, nucleosides and fatty acids, while the remaining 14 were matched to known ‘unknowns’, compounds that are routinely detected but have not been positively identified. These ‘unknowns’ were named according to their retention index followed by its base peak. Normalised to the internal standard, trimethylsilylated ribitol, the relative standard deviation (RSD) was 18% of the mean. 

**Figure 1:**
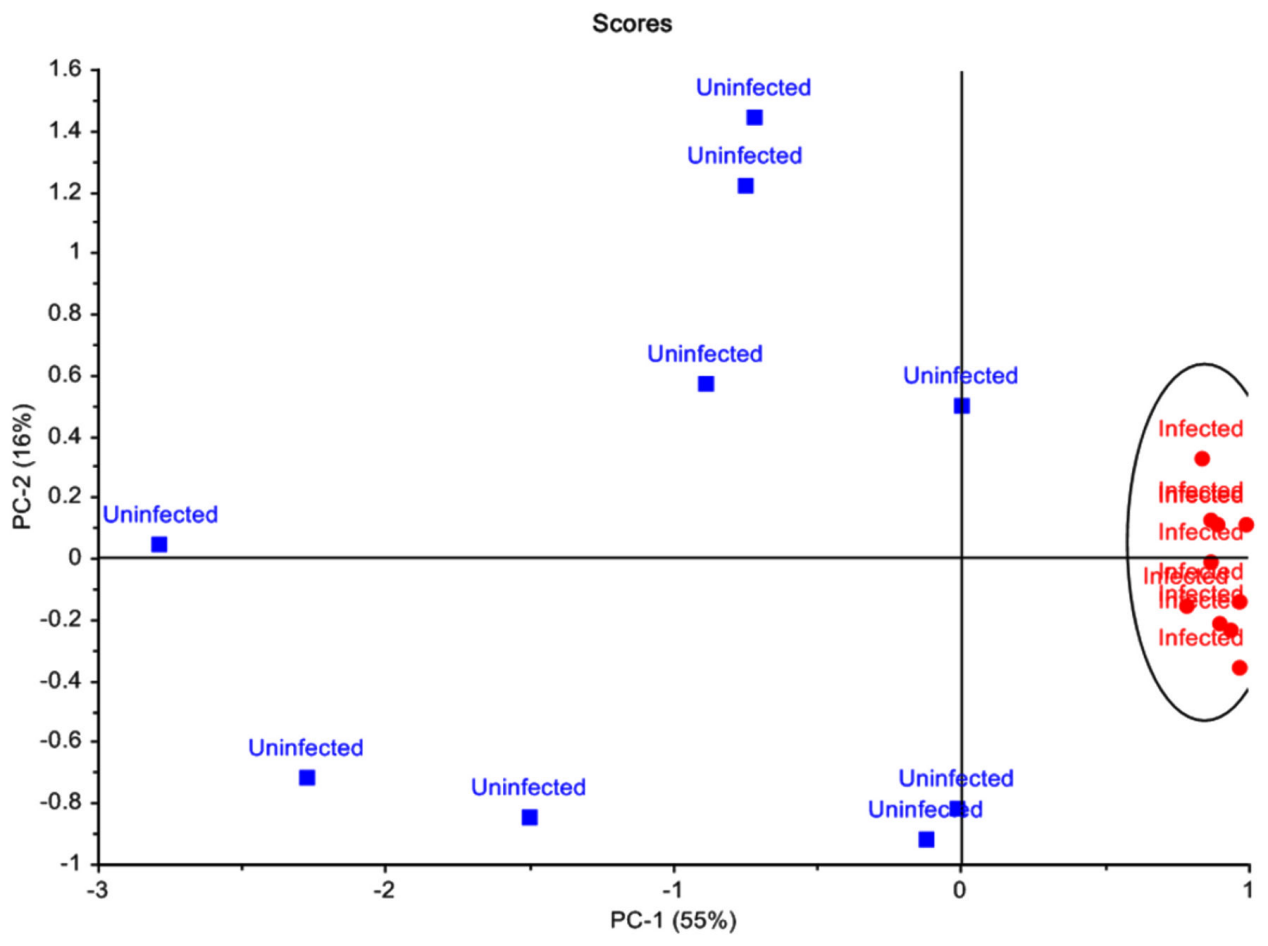
Principal component analysis of mice faecal metabolite profiles. Score plots showed distinct clustering patterns of metabolite profiles of *C. parvum* infected mice and the uninfected control mice, differentiating the two groups. *Cryptosporidium* infected mouse samples are represented by ● and uninfected control mouse samples are represented by ■.

**Figure 2:**
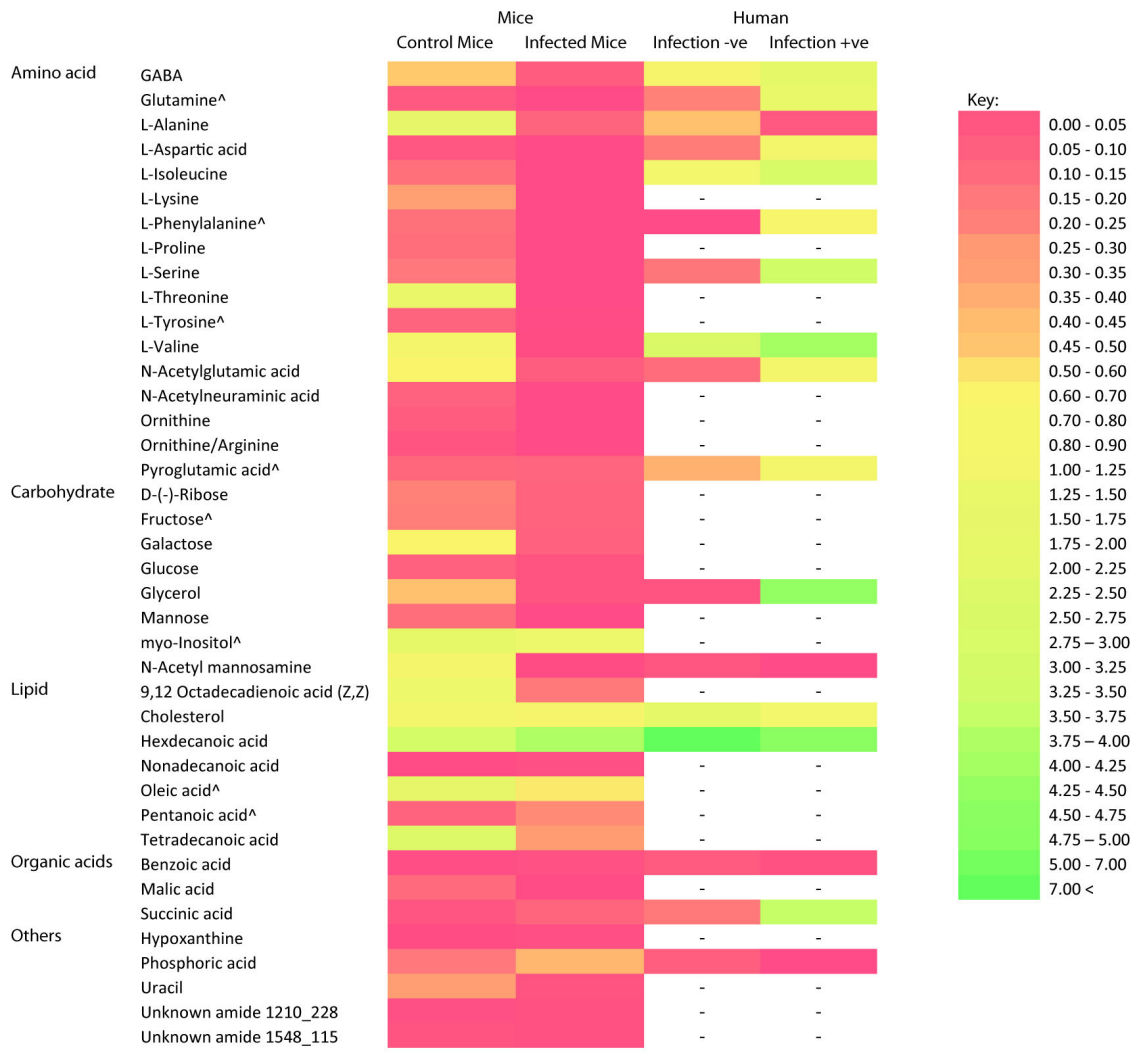
Faecal metabolites that contributed to the variance between infected and uninfected mice. Metabolites contributing to the variance between faecal metabolite profiles of mice infected with *C. parvum* and the uninfected control mice were compared to metabolites which contributed to the variance of faecal metabolite profiles of humans infected and not infected with *Cryptosporidium* from Ng et al., (2012). Heat map was generated using the mean of normalized peak area of the metabolite. All mice faecal metabolites showed statistically significant (p < 0.05) contributions to the variance except for those denoted by ^, where p > 0.05.

Principal component analysis (PCA) of the MS data normalised to the internal standard showed distinct clustering of the *C. parvum* infected mice and the uninfected control mice. The score plot generated showed that the infected mice samples clustered closely together, whereas samples from uninfected mice showed variation within the group (Figure 1). Principal component 1 (PC1) explained 55% of the variance, with PC2 explaining 16% of the variance observed. Based on the X-loadings, a total of 40 compounds, or metabolites that contributed most to the variance between the two groups were identified. These compounds, or metabolites, included amino acids (n = 17), carbohydrates (n = 8), lipids (n = 7), organic acids (n = 3), and metabolites from various other compound classes (n = 5) (Figure 2). Statistical analysis revealed that 33 of the 40 metabolites showed significant differences in levels (p > 0.05) between the group of infected and uninfected mice (Figure 2). Levels of metabolites observed in the infected mice were generally lower, when compared to the uninfected mice, with a mean ratio of 1:3.9. 

### Metabolites identified in faecal samples of *C. parvum* infected mice and uninfected mice

Levels of the 17 amino acids that contributed most to the variance between the mice groups were lower in the infected mice when compared to the uninfected mice (Figure 2). In addition, L-lysine, L-phenylalanine, glutamine, ornithine and N-acetylneuraminic acid were not detected in the infected mice samples. Mean levels of amino acids between infected and uninfected mice showed significant differences (p < 0.05), with the exception of L-tyrosine, L-phenylalanine, glutamine and pyroglutamic acid, which although detected in lower amounts in the infected mice compared to the uninfected mice, were not statistically different (p > 0.05). 

 The 8 carbohydrates which contributed to the variance included the monosaccharides, galactose, mannose, fructose, and glucose; the sugar alcohols, glycerol and myo-inositol and an amino sugar, N-acetylmannosamine (Figure 2). In the infected mice, lower levels of these carbohydrates were detected compared to the uninfected mice with significant differences (p < 0.05) in the levels detected, except for the levels of fructose, myo-inositol and glucose, which were not significantly different (p > 0.05).

For the lipids, significant differences (p < 0.05) were observed between the infected and uninfected mice groups with higher levels of hexadecanoic acid (ratio 1.69:1) and nonadecanoic acid (ratio 3.12:1), and lower levels of 9, 12-octadecadienoic acid (1:5.1), tetradecanoic acid (ratio 1:6.2) and cholesterol (1:1.43) detected in the infected mice group. Other lipid metabolites which contributed to the variance between the mice groups included pentanoic acid and oleic acid. Although higher levels of pentanoic acid (2:1) and lower levels of oleic acid (1:2.5) were detected in the infected mice compared to the uninfected mice, the differences were not significant (p > 0.05).

The 3 organic acids that contributed most to the variance between infected and uninfected mice groups showed significant differences in the levels detected (p < 0.05). Lower levels of malic acid were detected in the infected mice (ratio of 1:30), whereas for succinic acid and benzoic acid, higher levels were detected in the infected mice with a ratio of 2.56:1 and 1.72:1, respectively. 

Levels of the pyrimidine uracil were significantly lower (p < 0.05) in the infected mice with a ratio of 1:7.38. There was no statistical difference (p > 0.05) in the levels of hypoxanthine, detected, which was 1.5 times higher in the infected mice compared to the uninfected mice. Comparison of the two unknown compounds that contributed to variance between the infected and uninfected mice groups against the NIST library revealed chemical structure matches to the amide class of compounds. The first unknown amide (with a retention index of 1210 and base peak of 228), was detected in higher levels in the infected mice (p < 0.05), whereas there was no statistical difference between the levels of the second unknown amide (with a retention index of 1548 and base peak of 115) in the two mice groups. 

## Discussion

The mechanism of invasion of *Cryptosporidium*, from its initial attachment to enterocytes to subsequent development of an intracellular yet extracytoplasmic parasitophorous vacuole, results in changes to the physiological structure of the small intestinal luminal brush border, impairing mucosal absorption and permeability [14,15]. With impaired intestinal absorption and permeability due to the infection, studies conducted have shown a reduction in the uptake of nutrients such as glucose, amino acids, lipids and vitamin A [16–18]. It can be hypothesised that, with a reduction in uptake, these nutrients would be excreted, and that a higher abundance of metabolites would be detected in the faeces of *Cryptosporidium* infected mice. Results from the present study however, showed that this was not the case as analyses of the faecal metabolite profiles from both *Cryptosporidium* infected and uninfected mice showed that lower faecal metabolite abundances were generally recorded from the infected mice compared to that of the uninfected mice. 

Genome sequencing and biochemical data has revealed that *Cryptosporidium* is highly reliant on its host/environment for nutrients, as it is missing key metabolic pathways and lacks the ability for *de novo* synthesis of nucleosides, fatty acids and amino acids [19–23]. Hence, to supplement its streamlined metabolism, *Cryptosporidium* scavenges nutrients from the host intestinal lumen which, in effect, may have resulted in the lower faecal metabolite abundances detected in the infected mice in the present study. Although metabolites identified were synonymous between the two groups, metabolites that were found to contribute to the variance between the *Cryptosporidium* infected and uninfected mice included precursors to amino acids, carbohydrates, fatty acids, as well as derivatives of purine and pyrimidine metabolism, hypoxanthine and uracil. 

A total of 17 different amino acids detected in the mice faecal extracts contributed to the variance between the infected and uninfected mice. Abundance of these amino acids was significantly lower in the infected mice compared to the uninfected mice (p < 0.05), except for L-tyrosine, L-phenylalanine, glutamine and pyroglutamic acid, which showed no significant difference in mean abundance (p > 0.05) (Figure 2). The lower abundance of all 17 amino acids identified from the infected mice compared to uninfected may be a result of amino acid scavenging by *Cryptosporidium* from the intestinal lumen, as it has at least 11 amino acid transporters to enable scavenging from the host to supplement for its lack of ability to synthesize amino acids [19]. Although *C. parvum* shows capability in interconverting a number of amino acids, for example serine and glycine interconversion, conversion of aspartate to asparagine and reverse conversion of glutamate to glutamine [24], these amino acids were detected in lower abundance in the infected mice indicating that such interconversion may not have taken place. 

The highly streamlined metabolism of *Cryptosporidium*, which lacks genes encoding for mitochondria and tricarboxylic acid cycle enzymes, suggests that the parasite’s main energy pathway is glycolysis, where these mono- and poly- saccharides are scavenged and used as fuel for energy production [19,24]. This may have resulted in lower abundances of metabolites and intermediaries involved in energy metabolism being detected in infected mice when compared to uninfected mice. These metabolites include the complex sugar mannose, the monosaccharide glucose, glycerol and malic acid, which suggest an increase in both glycolysis and gluconeogenesis. The higher abundance of succinic acid, an intermediate of the tricarboxylic acid cycle (TCA), detected in both *Cryptosporidium* infected mice and humans, suggests a decrease in TCA activity during an infection [12]. This could be caused by an increase in demand for the TCA intermediates, oxaloacetate and α-ketoglutarate, to act as precursors for amino acid synthesis. This higher abundance of succinic acid may also result in a shift towards anaerobic glycolysis in the intestine as in the absence of oxygen, high levels of succinic acid may also inhibit the tricarboxylic acid cycle [12,25].

Despite only a narrow difference in ratio of benzoic acid abundance detected in *Cryptosporidium* infected mice and uninfected mice, the level of benzoic acid detected from the infected mice was significantly higher compared to the uninfected mice (p < 0.05) and contributed to the variance between these two groups. This was also observed in *Cryptosporidium* infected humans where higher abundance of benzoic acid was detected compared to the uninfected humans [12]. In mammals, benzoic acid is absorbed in the small intestine where it binds a conjugate with glycine, and is excreted as hippuric acid in the urine [26,27]. With *Cryptosporidium*’s dependency on the host for amino acids, glycine, which was detected in lower abundance in infected mice, may not have been available to conjugate with benzoic acid to supplement the metabolic demand from the infection, resulting in a decrease in acylation of benzoic acid. This could be confirmed by examining urine samples from infected mice for hippuric acid abundance, which should be lower in infected mice compared to uninfected mice.

 The faecal metabolite profile of lipids detected from both mouse groups in the present study consisted mainly of saturated fatty acids of 14 to 18 carbons in length, which play important roles in energy metabolism and cell membrane biosynthesis. Studies into the lipid metabolism of *C. parvum* revealed that the parasite lacks the ability to synthesise fatty acids *de novo*, but is capable of fatty acid chain elongation via the enzyme elongase CpLCE1, which is involved in elongating carbon C_12:0_ to C_16:0_ saturated fatty acid substrates to C_18:0_ products [24,28]. In the study conducted by Fritzler et al. [28], it was observed that when the C_14:0_ fatty acid was used as a substrate, the longest elongation product synthesised was C_16:0_ fatty acid, which then did not serve as a substrate for further elongation. This may explain for the lower abundance of tetradecanoic acid (C_14:0_), which could have been used as a substrate of elongase, resulting in the higher abundance of hexadecanoic acid (C_16:0_) but lower abundance of octadecanoic acid (C_18:0_), as there was no further elongation of C_16:0_ fatty acid substrates synthesized from C_14:0_ in the infected mice. The lower levels of cholesterol detected in the infected mice compared to uninfected mice was similar to that observed in the human faecal metabolite profile where levels of cholesterol were lower in those infected with *Cryptosporidium* compared to those uninfected (Table S1) [12]. Important to the development and life cycle of the parasite, a recent study showed that *Cryptosporidium* scavenges cholesterol from the intestinal lumen and enterocytes of its host [29], which may explain for the lower levels of cholesterol detected in both infected mice and humans. 

Of the metabolites that contributed most to the variance between *Cryptosporidium* infected and uninfected humans in the previous study by Ng et al. [12] and mice in this present study, the metabolites L-alanine, L-aspartic acid, L-valine, L-isoleucine, L-serine, L-phenylalanine, N-acetylglutamic acid, glutamic acid, pyroglutamic acid, δ-aminobutyric acid (GABA), N-acetyl mannosamine, glycerol, cholesterol, hexadecanoic acid, benzoic acid, succinic acid and phosphoric acid were identified in both faecal metabolite profiles of humans and mice. In the previous study however, higher levels of metabolites were generally detected in *Cryptosporidium* positive patients, differing from the results of the present study, where lower metabolite levels were generally detected in faecal samples from *Cryptosporidium* infected mice (Table S1). Differences in metabolite profiles between different host types have been previously reported by Saric et al. [30]. In that study, a comparison of faecal metabolite profiles from mice, rats and humans showed that the levels of metabolites differed between the species, presumably as a result of different endogenous and exogenous perturbations and differences in the gut microbiome between species [30]. Despite the differences in faecal metabolite profiles between *Cryptosporidium* infected humans and mice, metabolomic analysis in both studies was still able to clearly differentiate between infected and uninfected hosts, as well as provide information on the metabolic activity of the parasite during the infection based on faecal metabolite profiles. 

Metabolites that contributed to the differences between infected and uninfected groups in both the present study and Ng et al. [12] are key nutrients scavenged by *Cryptosporidium* to supplement its streamlined metabolic pathway, which is supported by results from recent *Cryptosporidium* transcriptomic studies. These studies have shown that genes that annotated for transporters and metabolic enzymes for key nutrients (sugars, nucleotides, amino acids and lipids) were highly expressed at the time of infection [23,31]. Although no metabolite unique to *Cryptosporidium* infection was identified in the present study, the abundance of metabolites detected in the faeces of mice infected with *C. parvum* compared to uninfected mice reflects not only on the effects of the infection on the host environment where absorption capabilities in the small intestine have been impaired, but also the fate of metabolites that were not absorbed. 

Metabolomics may be useful for the diagnosis of *Cryptosporidium* infections, as it allows for detection based on differences as a result of physiological changes caused by the infection rather than detecting oocysts in faeces by microscopy or PCR, where sensitivity is limited by both the numbers of oocysts present and the intermittent shedding of oocysts. However, whether the profiles observed are unique to *Cryptosporidium* infection or are seen in infections with other gastrointestinal pathogens needs to be ruled out along with extensive analysis of the metabolites profiled before this method could be developed as a diagnostic tool. Metabolite profiling of *Cryptosporidium* infection should also be expanded to include profiles from other biofluids, such as blood and urine, as well as tissue samples, which will provide a more comprehensive understanding of *Cryptosporidium* host-parasite interaction and its impact on host metabolism. This could potentially result in the identification of unique metabolites as biological markers for better diagnosis and open new avenues for the development of anti-*Cryptosporidium* therapies.

## Supporting Information

Table S1 **Mean of normalized peak area of metabolites (compounds) identified in faecal samples of mice infected with *C. parvum* and the uninfected control mice compared to faecal metabolite profile of humans infected and not infected with *Cryptosporidium* from a previous study (Ng et al., 2012).**
(DOCX)
