# Supplementary material for: Metabolomic Profiling of Faecal Extracts from Cryptosporidium parvum Infection in Experimental Mouse Models
Source: PLoS One. 2013 Oct 18;8(10):e77803. doi: 10.1371/journal.pone.0077803 (PMC3800111; doi:10.1371/journal.pone.0077803)
Supplement: Table S1 — Mean of normalized peak area of metabolites (compounds) identified in faecal samples of mice infected with C. parvum and the uninfected control mice compared to faecal metabolite profile of humans infected and not infected with Cryptosporidium from a previous study (Ng et al., 2012). (DOCX) [file pone.0077803.s001.docx]

Table S1: Mean of normalized peak area of metabolites (compounds) identified in faecal samples of mice infected with *C. parvum* and the uninfected control mice compared to faecal metabolite profile of humans infected and not infected with *Cryptosporidium* from a previous study (Ng et al., 2012).

| **Metabolites** | **Mice** | | **Human** | |
| --- | --- | --- | --- | --- |
|  | **Uninfected** | **Infected** | **Uninfected** | **Infected** |
| 9,12 Octadecadienoic acid (Z,Z) | 1.02857 | 0.20211 |  |  |
| 9-Octadecenamide (Z) | 0.00000 | 0.00266 |  |  |
| 9-Octadecenenitrile (Z) | 0.00000 | 0.00361 |  |  |
| Alanine | 0.09813 | 0.00000 |  |  |
| Arabinose | 0.06795 | 0.01505 |  |  |
| Aspartic acid | 0.03258 | 0.04954 |  |  |
| Azelaic acid | 0.00345 | 0.01257 |  |  |
| Benzoic acid | 0.01703 | 0.02933 | 0.07116 | 0.03384 |
| beta-Alanine | 0.00028 | 0.00116 |  |  |
| Cellobiose | 0.00000 | 0.00028 |  |  |
| Cholesterol | 0.83152 | 0.75479 | 1.29944 | 0.85916 |
| D-(-)-Ribose | 0.23501 | 0.11145 |  |  |
| D-Pinitol | 0.00194 | 0.00281 |  |  |
| Fructose | 0.22061 | 0.11492 | 0.00279 | 2.76036 |
| Fructose | 0.11235 | 0.06616 |  |  |
| Fumaric acid | 0.01766 | 0.00036 |  |  |
| GABA | 0.47900 | 0.08688 | 0.75856 | 1.29266 |
| Galactose | 0.62402 | 0.10529 |  |  |
| Galactose | 0.01600 | 0.00056 |  |  |
| g-Hydroxyglutaric acid | 0.00000 | 0.00071 |  |  |
| Glucopyranose | 0.00000 | 0.00015 |  |  |
| Glucose | 0.10210 | 0.03804 |  |  |
| Glucose | 0.76671 | 0.34329 |  |  |
| Glucuronic acid | 0.00150 | 0.00000 |  |  |
| Glutamine | 0.06168 | 0.00000 |  |  |
| Glutaric acid | 0.00020 | 0.00000 |  |  |
| Glyceric acid | 0.03987 | 0.00074 |  |  |
| Glycerol | 0.45593 | 0.04332 | 0.03898 | 4.36919 |
| Glycerol 3-Phosphate | 0.03289 | 0.03980 |  |  |
| Hexdecanoic acid | 1.88391 | 3.21622 | 7.12470 | 4.80394 |
| Hypoxanthine | 0.01141 | 0.01946 |  |  |
| Lactose | 0.00000 | 0.00028 |  |  |
| L-Alanine | 1.19349 | 0.11553 | 3.07196 | 4.41755 |
| L-Alanine | 0.01213 | 0.00189 | 0.45676 | 0.07358 |
| L-Asparagine | 0.00037 | 0.00000 |  |  |
| L-Aspartic acid | 0.05682 | 0.00032 | 0.20789 | 0.81873 |
| L-Glutamic acid | 0.22357 | 0.12511 | 0.23186 | 1.18186 |
| L-Glycine | 0.19238 | 0.07508 |  |  |
| L-Isoleucine | 0.16561 | 0.00056 | 0.87763 | 1.75428 |
| L-Lysine | 0.34528 | 0.00000 |  |  |
| L-Methionine | 0.03741 | 0.00000 |  |  |
| L-Phenylalanine | 0.16680 | 0.00000 | 0.00000 | 0.69200 |
| L-Proline | 0.15992 | 0.00147 |  |  |
| L-Proline | 0.01467 | 0.00000 |  |  |
| L-Serine | 0.15947 | 0.00979 | 0.18630 | 2.00585 |
| L-Serine | 0.19654 | 0.00252 |  |  |
| L-Threonine | 1.11461 | 0.00230 |  |  |
| L-Threonine | 0.34061 | 0.00591 |  |  |
| L-Tyrosine | 0.11709 | 0.00564 |  |  |
| L-Valine | 0.79384 | 0.01195 | 1.65323 | 3.62800 |
| L-Valine | 0.26370 | 0.00141 |  |  |
| Lysine | 0.00000 | 0.00023 |  |  |
| Malic acid | 0.14626 | 0.00486 |  |  |
| Maltose | 0.00000 | 0.00156 |  |  |
| Mannose | 0.16015 | 0.00283 |  |  |
| myo-Inositol | 1.24033 | 1.03768 |  |  |
| myo-inositol | 0.04207 | 0.01482 |  |  |
| N-Acetyl mannosamine | 0.80440 | 0.01258 |  |  |
| N-Acetyl mannosamine | 0.38392 | 0.00470 | 0.04638 | 0.00000 |
| N-Acetylglutamic acid | 0.63399 | 0.08983 | 0.15029 | 0.83735 |
| N-Acetylneuraminic acid | 0.11003 | 0.00000 |  |  |
| Nonadecanoic acid | 0.00863 | 0.02710 |  |  |
| Octadecanoic acid | 1.60698 | 1.73383 |  |  |
| Oleic acid | 1.19663 | 0.57099 |  |  |
| Ornithine | 0.08115 | 0.00000 |  |  |
| Ornithine/Arginine | 0.04499 | 0.00000 |  |  |
| Pantothenic acid | 0.01983 | 0.00000 |  |  |
| Pentanoic acid | 0.11422 | 0.27086 |  |  |
| Phosphoric acid | 0.19807 | 0.41548 | 0.09436 | 0.00000 |
| Phosphoric acid | 0.00973 | 0.01107 |  |  |
| p-Hydroxybenzoic acid | 0.00264 | 0.00000 |  |  |
| Putrescine | 0.06820 | 0.06379 |  |  |
| Pyroglutamic acid | 0.13045 | 0.12352 | 0.40310 | 0.85106 |
| Siloxane | 0.03971 | 0.06942 |  |  |
| Sorbitol | 0.00219 | 0.00000 | 0.00000 | 0.68246 |
| Succinic acid | 0.04901 | 0.12651 | 0.20056 | 2.40465 |
| Sucrose | 0.00753 | 0.00387 | 0.00000 | 0.87304 |
| Tetradecanoic acid | 0.00132 | 0.00000 |  |  |
| Tetradecanoic acid | 1.53994 | 0.33194 |  |  |
| Thymine | 0.02137 | 0.00012 |  |  |
| Tryptamine | 0.00480 | 0.00000 |  |  |
| Tyramine | 0.04344 | 0.00177 |  |  |
| Uracil | 0.33669 | 0.04564 | 0.50018 | 0.77988 |
| Urea | 0.01857 | 0.01261 | 0.00000 | 5.49976 |
| Uric acid | 0.26681 | 0.00000 |  |  |
| Xylitol | 0.00457 | 0.00000 | 0.00181 | 0.00606 |
| Xylose | 0.03934 | 0.00432 | 0.02144 | 0.00000 |
| Unknown amide_1210_228 | 0.02602 | 0.03632 |  |  |
| Unknown_1270_174 | 0.43404 | 0.30429 |  |  |
| Unknown_1339_170 | 0.03800 | 0.07740 |  |  |
| Unknown_1410_234 | 0.00000 | 0.00256 |  |  |
| Unknown amide_1548_115 | 0.03724 | 0.03600 |  |  |
| Unknown_1616_142 | 0.03440 | 0.00000 |  |  |
| Unknown_1673_116 | 0.00057 | 0.00000 |  |  |
| Unknown_1708_173 | 0.00136 | 0.00000 |  |  |
| Unknown_1871_319 | 0.00000 | 0.00062 |  |  |
| Unknown_1943_318 | 0.00038 | 0.01056 |  |  |
| Unknown_1951_217 | 0.00065 | 0.00000 |  |  |
| Unknown_2021_204 | 0.00338 | 0.00079 |  |  |
| Unknown_2306_204 | 0.00662 | 0.00000 |  |  |
| Unknown_2333_217 | 0.00047 | 0.00000 |  |  |
